# Symptomatic quadrigeminal cistern lipoma

**DOI:** 10.11604/pamj.2015.20.328.6495

**Published:** 2015-04-07

**Authors:** Rachid Ammor, Assou Ajja

**Affiliations:** 1 Department of Neurosurgery, Military Hospital My Ismail, Meknes, Morocco

**Keywords:** Quadrigeminal cistern lipoma, mass lesion, neurovascular

## Image in medicine

A 55 year old man, with no history, referred by his doctor for headache lasting for 3 months, a week before the patient had two complex partial seizures became secondary generalized. Physical examination was unremarkable. EEG and laboratory data were normal. The CT scan showed a highly hypodense (-73 to -86 H.U) mass of the quadrigeminal region (A). The cranial MRI with and without contrast administration, revealed a non-enhancing 3×2,7cm quadrigeminal cistern mass lesion, hyperintense on T1 (B)and T2 (C) weighted sequences, On fat suppression pulse sequence intensity of the lesion is homogeneously decreased (D). These signal intensities were consistent with fat. The patient was put under valproic acid with a very good control without any seizure activity for 12 months. Intracranial symptomatic lipomas located in the quadrigeminal cistern are extremely rare. These lesions are usually managed conservatively as surgical removal is difficult owing to their deep localization and close contiguity with adjacent neurovascular structures.

**Figure 1:**
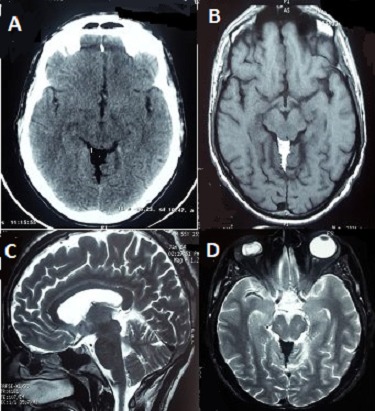
(A) CT scan showing a hypodense lesion in quadrigeminal plate cistern. (B,C,D) MRI scan, T1 imaging axial view: (B) T2 imaging sagittal view; (C) axial fat-suppression sequence; (D) quadrigeminal cistern lipoma

